# Agrimoniin, an Active Ellagitannin from *Comarum palustre* Herb with Anti-α-Glucosidase and Antidiabetic Potential in Streptozotocin-Induced Diabetic Rats

**DOI:** 10.3390/molecules22010073

**Published:** 2017-01-02

**Authors:** Nina I. Kashchenko, Nadezhda K. Chirikova, Daniil N. Olennikov

**Affiliations:** 1 Institute of General and Experimental Biology, Siberian Division, Russian Academy of Science, Sakh’yanovoy Street 6, Ulan-Ude 670047, Russia; ninkk@mail.ru; 2 Department of Biochemistry and Biotechnology, North-Eastern Federal University, 58 Belinsky Street, Yakutsk 677027, Russia; hofnung@mail.ru

**Keywords:** *Comarum palustre*, ellagitannins, α-glucosidase inhibition, anti-diabetic activity, agrimoniin

## Abstract

Naturally existing α-glucosidase inhibitors from traditional herbal medicines have attracted considerable interest to treat type 2 diabetes mellitus (DM). The present study aimed to evaluate the anti-α-glucosidase activity of extracts from marsh cinquefoil (*Comarum palustre* L.), their hypoglycaemic action and detection of the responsible compounds. A 60% ethanol extract from *C. palustre* herb revealed the highest inhibitory activity against α-glucosidase (IC_50_ 52.0 μg/mL). The HPLC analysis of the major compounds resulted in detection of 15 compounds, including ellagitannins, flavonoids, catechin and other compounds. Using HPLC activity-based profiling a good inhibitory activity of agrimoniin-containing eluates against α-glucosidase was demonstrated. The removal of ellagitannins from the *C. palustre* extract significantly decreased α-glucosidase inhibition (IC_50_ 204.7 μg/mL) due to the high enzyme-inhibiting activity of the dominant agrimoniin (IC_50_ 21.8 μg/mL). The hypoglycaemic effect of *C. palustre* extracts before and after ellagitannin removal, agrimoniin and insulin was evaluated on streptozotocin-induced experimental model. Diabetic rats treated with agrimoniin and *C. palustre* extract before ellagitannin removal showed significant increases in the levels of plasma glucose and glycosylated hemoglobin and significant decreases in the levels of plasma insulin and hemoglobin. The data obtained confirm the leading role of agrimoniin in the antidiabetic activity of the herb *C. palustre* and allows us to suggest the use of this plant as a possible dietary adjunct in the treatment of DM and a source of new oral hypoglycaemic agents.

## 1. Introduction

Diabetes mellitus (DM), a chronic metabolic disorder, has become a worldwide health problem. According to a report of the World Health Organization, approximately 422 million people worldwide suffered from DM in 2014 compared to 108 million in 1980. The global prevalence (age-standardized) of diabetes has nearly doubled since 1980, rising from 4.7% to 8.5% in the adult population [1]. DM is characterized by persistent hyperglycaemia following disfunctions in insulin emission, insulin activity or both. High blood sugar can produce long-term complications such as cardiovascular and renal disorders, retinopathy and poor blood flow [2]. People who develop type 2 diabetes pass through a phase of impaired glucose tolerance (IGT) which is characterized by defects in the action and/or secretion of insulin. Any intervention in the IGT phase that reduces resistance to insulin or protects the β-cells, or both, should prevent or delay progression to diabetes. The inhibition of α-glucosidase, a key intestinal enzyme involved in the digestion of carbohydrates is one of the possible ways to avert diabetes. Its inhibition causes a decrease in both postprandial hyperglycaemia and hyperinsulinaemia, and thereby may improve sensitivity to insulin and alleviate the stress on β-cells [3]. 

Hypoglycemic synthetic α-glucosidase inhibitors exemplified by acarbose have been approved for clinical use in control of type 2 diabetes. However, the application of such remedies is complicated by adverse gastrointestinal side-effects (abdominal discomfort, flatulence and diarrhoea) and may limit long-term compliance to therapy [4]. In addition, acarbose was not well tolerated in children with idiopathic postprandial hyperinsulinaemic hypoglycaemia [5]. Thus, naturally existing α-glucosidase inhibitors have attracted considerable interest for treating DM.

Medicinal plants are gradually gaining global acceptability given their potential as a source of bioactive agents with potential applications as pharmaceuticals. An antidiabetic plant compound could exert a beneficial effect in the diabetic environment by improving or mimicking insulin action and/or by enhancing insulin secretion [6]. There is rising evidence that polyphenolic compounds from different plant families have potent inhibitory activity against α-glucosidase and are responsible for the lowering of blood glucose [7,8,9].

The family Rosaceae comprises around 110 genera and about 4830 species. Rosaceous plants with purported anti-diabetic properties have been used in folk medicine, traditional healing systems, and as complementary and alternative medicine. Several Rosaceous plant objects have been proved to inhibit α-glucosidase including *Vauquelinia corymbosa* [10] and *Prunus domestica* [11]. Recently a lot of attention is paid to members of the Rosoideae subfamily, due to their high α-glucosidase inhibiting activity, in particular, representatives of the genus Rubus—*R. fruticosus* (blackberry) [12] and *R. idaeus* [13].

Marsh cinquefoil (*Comarum palustre* L.) is another member of the Rosoideae subfamily. It is a low-growing herbaceous plant, found in temperate zones around the globe and most commonly in damp, marshy places in Europe, Asia and Northern America. The aqueous extracts of *C. palustre* and its roots are widely used in Siberian folk medicine for the treatment of a variety of human diseases such as hypertension, rheumatic arthritis, ischemic heart diseases, etc. [14]. Moreover, there are data about application of this plant species for the treatment of DM and its use as a hypoglycaemic remedy [15]. The known literature data on chemical composition and biological activities relates primarily to the pectic polysaccharide comaruman isolated from the aerial parts and its anti-inflammatory activity [16,17], confirming the poor general knowledge level of chemical and pharmacological data of this plant species, and suggesting a need for further work in this direction.

The present research, now in its initial stages, is aimed at investigation of the highest inhibitory effect on α-glucosidase of aqueous and ethanol extracts from different parts of *C. palustre* (herb, roots, flowers, seeds), further detection the most active compounds responsible for the manifestation of anti-α-glucosidase activity with the use of HPLC activity-based profiling and revealing the antidiabetic potential of these compounds and extracts in a streptozotocin-induced diabetic model in rats.

## 2. Results and Discussion

### 2.1. Inhibition of α-Glucosidase by *C. palustre* Extracts

As inhibition of α-glucosidase is believed to be one of the most effective approaches for diabetes care, we decided to investigate this activity of *C. palustre* extracts. According to numerous studies water and ethanol are the most commonly used extractants for such investigations [18,19,20]. Due to the absence of common recommendations on the choice of extractant, water and ethanol were used. At the preliminary stage of the study we used aqueous (decoction, infusion) and ethanol extracts (tincture, 30%, 60%, 96% extracts) from herb, roots, flowers and seeds of *C. palustre* and examined their inhibitory effect on α-glucosidase (Table 1).

Investigation of the influence of extracts from *C. palustre* demonstrated the good inhibition of α-glucosidase by the extracts of herb and flowers relative to the seeds and roots. There were no significant differences between aqueous extracts: decoction and infusion from the herb of *C. palustre* revealed similar α-glucosidase inhibition values (IC_50_ 127.4 and 142.9 μg/mL, respectively).

Aqueous extracts from roots, flowers and seeds displayed no inhibition of α-glucosidase activity up to a concentration >300 μg/mL while their ethanol extracts possessed moderate activity. The most active was a 60% ethanol extract from the herb which revealed the highest activity (IC_50_ 52.0 μg/mL); flower extract was weaker (IC_50_ 104.2 μg/mL) and roots were even less active (IC_50_ 272.7 μg/mL). In all extraction cases, extracts from *C. palustre* herb were more active than acarbose (IC_50_ 294.4 μg/mL), a known α-glucosidase inhibitor used as a positive control. Based on the anti-α-glucosidase activity results of *C. palustre* extracts we can confirm the potential of 60% extract from *C. palustre* herb as a good source of α-glucosidase inhibitors.

### 2.2. Screening of α-Glucosidase Inhibitors from *C. palustre* Herb Extract

**Figure 1:**
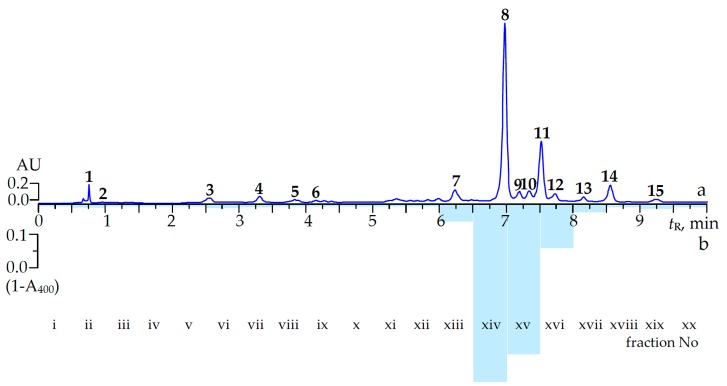
The microcolumn reversed phase HPLC chromatogram of (a) *C. palustre* extract at 270 nm and (b) (1-A_400_) values of fraction (i–xx) after post-column derivatization. Compounds: **1**. 2-pyrone-4,6-dicarboxylic acid; **2**. gallic acid; **3**. *α*-pedunculagin; **4**. *β*-pedunculagin; **5**. procyanidin B3; **6**. (+)-catechin; **7**. potentillin; **8**. agrimoniin; **9**. ellagic acid; **10**. rutin; **11**. miquelianin; **12**. isoquercitrin; **13**. nicotiflorin; **14**. astragalin; **15**. afzelin. AU: absorption units; *t*_R_: retention time.

**Figure 2:**
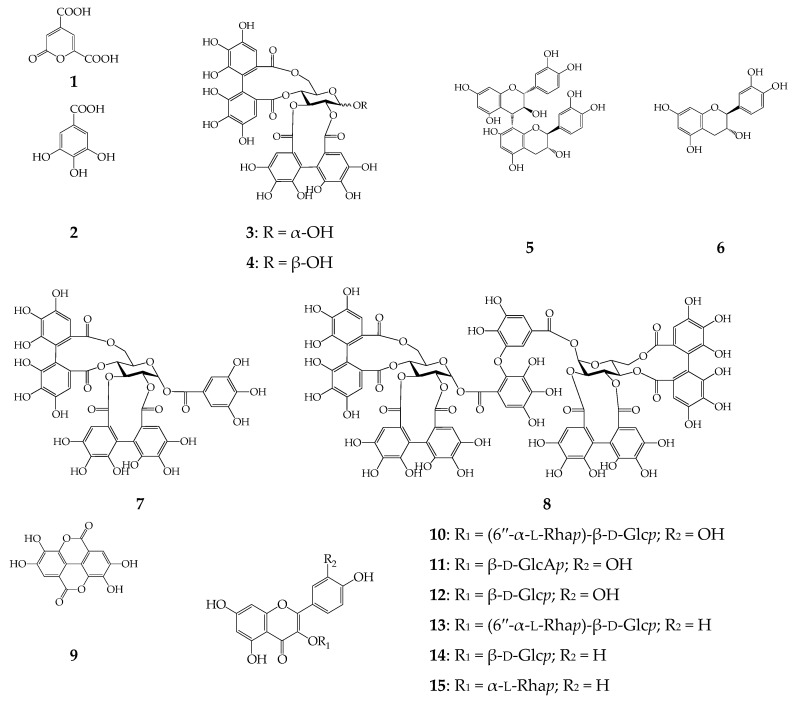
Chemical structures of compounds **1**–**15** detected in *C. palustre* extract. β-d-Glcp—β-d-glucopyranose; β-d-GlcAp—β-d-glucuronopyranose; α-l-Rhap—α-l-rhamnopyranose.

In order to characterize the chemical profile of 60% ethanol extract of *C. palustre* herb a previously described microcolumn reversed phase HPLC procedure with UV and MS detection (MC-RP-HPLC-UV-MS) was applied (Figure 1a) [21]. The mass spectral data of the *C. palustre* herb extract were obtained in negative ion mode, which provided more structural information than those obtained in positive ion mode. The data related to retention time (tR), UV spectra and ESI-MS data are summarized in Table 2. By comparing the data obtained with those of isolated compounds or commercial standards, 15 constituents were identified, four ellagitannins (α-pedunculagin, β-pedunculagin, potentillin, agrimoniin), six flavonoids (rutin, miquelianin, isoquercitrin, nicotiflorin, astragalin, afzelin), one catechin ((+)-catechin), 2-pyrone-4,6-dicarboxylic acid, gallic acid, procyanidin B3 and ellagic acid (Figure 2). All mentioned compounds were isolated earlier from *C. palustre* herb of Siberian origin [21].

To identify the compounds of interest of *C. palustre* herb with high α-glucosidase inhibitory activity, the extract investigated was submitted to HPLC activity-based profiling. This technique is a miniaturized and highly effective approach for localization and characterization of bioactive natural products with minute amounts of injected extracts [22,23,24]. This technique combines the speed and separation power of HPLC with the structural information of online spectroscopy and miniaturized bioassays [25]. For detection of anti-α-glucosidase inhibitors in *C. palustre* herb extract the procedure of small-scale semi-preparative microfractionation by reversed-phase HPLC was used. This yielded 20 microfractions of 30 s each that were transferred to a deep-well microtitre plate. Then microfractions were dried, redissolved in buffer solution and enzyme solution was added to evaluate eluate inhibition of α-glucosidase. The optical densities at 400 nm (A_400_) as (1-A_400_) values of microfractions after post-column derivatization are shown in Figure 1b. Major inhibition was observed in the time window of 6.30 to 8.00 min, corresponding to fractions xiv–xvi which displayed the highest anti-α-glucosidase activity potential with (1-A_400_) values of 0.53, 0.44 and 0.12, respectively, while the activities of other fractions were not significantly different from zero.

Correlation of the (1-A_400_) data of eluates with MC-RP-HPLC-UV-MS characterization of the components allowed us to distinguish two well-separated major and three minor peaks with close retention times: agrimoniin (**8**) at 6.98 min, ellagic acid (**9**) at 7.20 min, rutin (**10**) at 7.33 min, miquelianin (**11**) at 7.50 min and isoquercitrin (**12**) at 7.72 min. The highest inhibition of α-glucosidase in fraction xiv was due to the presence of a single compound, agrimoniin, which belongs to the ellagitannins.

The inhibitory effectiveness of ellagitannins against α-glucosidase has been shown previously [26,27]. However, the presence of four substances (ellagic acid, rutin, miquelianin, isoquercitrin) in the two subsequent fractions did not provide a clear answer to the question about the source of the inhibitory activity. According to the literature data, ellagic acid showed almost no inhibition against α-glucosidase [28,29], while flavonoids can be effective in inhibiting α-glucosidase activity—a total of 103 flavonoids reported in the literature showed glucosidase inhibitory activity [30].

However, McDougall et al. showed that various phenolic compounds may influence different steps in digestion in a synergistic manner—deletion from the extract of ellagitannins with previously proven high inhibiting activity against α-glucosidase finally retarded inhibition of the enzyme [31]. In this way, we decided to remove ellagitannins from *C. palustre* herb extract to find out how it would affect the inhibition of α-glucosidase.

### 2.3. Effect of Ellagitannin Remove from *C. palustre* Extract on α-Glucosidase Inhibition

Quantification of the main phytochemicals in *C. palustre* extract (named extract A) was realised by a previously described MC-HPLC-UV procedure [21]. The results showed that the total content of quantifiable compounds in *C. palustre* extract was 488.56 mg/g (Table 3). The highest content, 284.35 mg/g, was detected for ellagitannins with agrimoniin as the dominant compound (240.94 mg/g). The total concentration of the flavonoids was 135.75 mg/g with miquelianin and astragalin as main compounds present at 80.81 and 25.40 mg/g, respectively. Detectable amounts of (+)-catechin and procyanidin B_3_ were found in *C. palustre* extract, with values 28.02 and 30.02 mg/g, respectively. Some components such as gallic acid, 2-pyrone-4,6-dicarboxylic acid and ellagic acid were minor. The removal of ellagitannins from *C. palustre* extract was realized with the use of a polyamide solid-phase extraction (SPE) procedure. Ultimately, a detannified extract (named extract B) was obtained. Quantification of the phenolic components in the extract B demonstrated the absence of gallic acid, α-pedunculagin, β-pedunculagin, (+)-catechin, potentillin and ellagic acid which were bound to the polyamide sorbent. The total ellagitannin content in the B-extract was 1.03 mg/g and the total concentration of flavonoids was 196.99 mg/g. The results of HPLC quantification are clear evidence that the extract B is adetannifed extract poor in ellagitannins.

**Figure 3:**
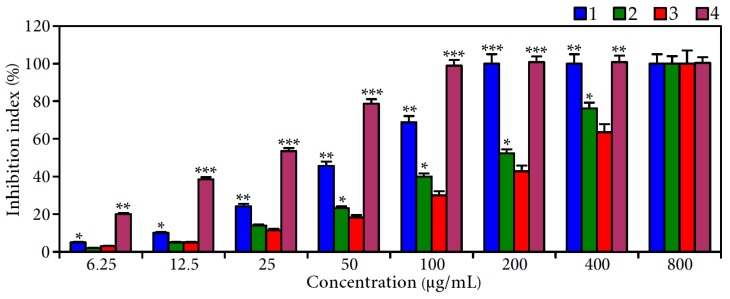
α-Glucosidase inhibition of *C. palustre* extracts A (**1**); and B (**2**); acarbose (**3**); and agrimoniin (**4**). Values shown are mean ± SD (*n* = 3). Asterisks indicate statistically significant differences from acarbose (* *p* < 0.05; ** *p* < 0.01; *** *p* < 0.001).

In order to evaluate the anti-α-glucosidase activity of extract A, B and agrimoniin, the most active enzyme inhibitor according our screening results, the α-glucosidase inhibitory activity of related extracts and compounds was estimated using in vitro assays (Figure 3). The IC_50_ values of extracts A and B were 52.0 and 204.7 μg/mL, respectively, demonstrating the most potent α-glucosidase inhibition of *C. palustre* herb extracts before tannin removal. Comparing the activity of acarbose as a reference compound (IC_50_ 294.4 μg/mL), the data obtained clearly demonstrated high inhibiting activity of *C. palustre* herb extract. The enzyme inhibition parameter of the dominant agrimoniin was 21.8 μg/mL, confirming its leading role in the anti-α-glucosidase activity of *C. palustre* extract.

According to literature data, flavonoids with high anti-α-glucosidase activity usually possess the following structural features: (1) the presence of a C2=C3 double bond in ring C; (2) a dihydroxyl group in ring B; and (3) the presence of C-5 and C-7 hydroxyl groups in ring A; one of the most prominent flavonols is quercetin [32]. From the screening results, all potentially active flavonoids in the *C. palustre* herb extract—inhibitors of α-glucosidase (miquelianin, isoquercitrin, rutin)—relate to glycosides of quercetin and possess close chemical structures. Miquelianin and isoquercitrin are monoglycosides of quercetin while rutin is its diglycoside. It has previously been shown that glycosylation of flavonols obviously weakens their inhibitory activity on α-glucosidase [33]. Li et al. compared the effects of quercetin, isoquercetin and rutin as α-glucosidase inhibitors with IC_50_ values of 0.017, 0.185 and 0.196 mM/L, respectively, compared with acarbose’s IC_50_ of 0.091 mM/L. The inhibitory activity of quercetin glycosides decreased more than 10-fold compared with the aglycone [34]. Also, it was shown that monoglycosides of flavonoids are better inhibitors of α-glucosidase than their polyglycoside forms [35,36], so it can be concluded that the inhibitory activity of miquelianin and isoquercitrin on α-glucosidase was close and weak; rutin possessed the worst inhibitory activity in relation to this enzyme.

In turn, the high inhibitory activity of ellagitannins dominant in *C. palustre* herb extract A relative to α-glucosidase could be explained by a specific property such as the ability to precipitate some proteins. Protein precipitation occurs as a surface phenomenon in which the phenolic groups of tannins bind to the protein surface [37]. Concerning the principle of association between polyphenols and protein at low concentrations, it was suggested that polyphenols bind to one or more sites of the protein surface and form a hydrophobic monolayer, which then causes aggregation and precipitation. In contrast, at high protein concentrations, polyphenols make complexes and cross-link with the protein and, by this multiple interaction, they form a hydrophobic surface and thus elicit precipitation [38]. The mechanism of the inhibitory activity against α-glucosidase of ellagitannins from the extract analysed could be tannin–protein binding which makes changes to the conformation of the enzyme and reduces α-glucosidase activity by association and precipitation.

### 2.4. Antidiabetic Effect of *C. palustre* Extracts and Agrimoniin in Stretpozotocin Induced Diabetic Rats

There is increasing evidence that polyphenolic compounds, especially ellagitannins, from plants can cause insulin-like effects on glucose utilization [39,40,41]. Earlier, the ability of dimeric ellagitannins from strawberry extract to reduce elevated blood glucose was shown [42]. In this connection, we decided to estimate the hypoglycaemic activity of *C. palustre* extracts (A and B) and evaluate the same activity for the predominant dimeric ellagitannin agrimoniin. The experimental diabetic model used was type 2 since low dose of diabetogenic agent streptozotocin (STZ; 60 mg/kg) partly destroys the beta cells of the islets of Langerhans bringing about inadequate insulin discharge, creating type 2 diabetes [43]. An insufficient release of insulin causes hyperglycaemia, which results in oxidative damage by the generation of reactive oxygen species and development of diabetic complications [44]. It is the animal model generally used for diabetic studies [45].

**Figure 4:**
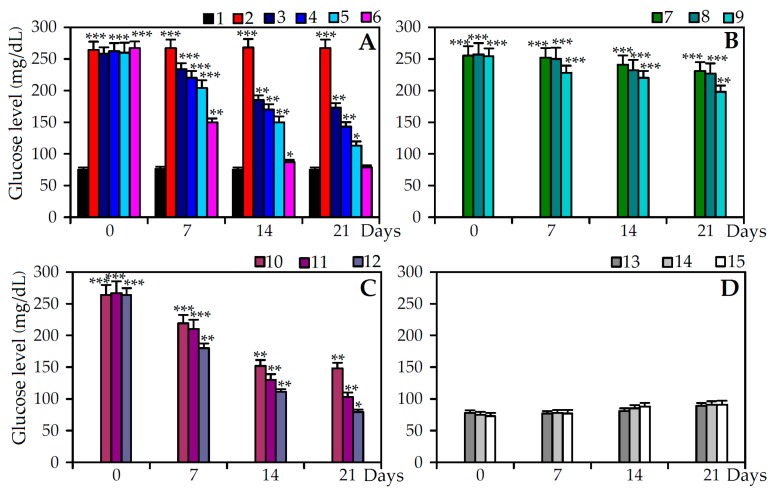
Fasting blood glucose level response in diabetic (STZ) and non-diabetic rats. (**A**) **1**. Control; **2**. STZ + saline; **3**. STZ + Extract A (100 mg/kg); **4**. STZ + Extract A (200 mg/kg); **5**. STZ + Extract A (400 mg/kg); **6**. STZ + Insulin; (**B**) **7**. STZ + Extract B (100 mg/kg); **8**. STZ + Extract B (200 mg/kg); **9**. STZ + Extract B (400 mg/kg); (**C**) **10**. STZ + Agrimoniin (25 mg/kg); **11**. STZ + Agrimoniin (50 mg/kg); **12**. STZ + Agrimoniin (100 mg/kg); (**D**) Non-diabetic rats: **13**. Extract A (400 mg/kg); **14**. Extract B (400 mg/kg); **15**. Agrimoniin (100 mg/kg). Values shown are mean ± SD (*n* = 6–8). Asterisks indicate statistically significant values from control (* *p* < 0.05; ** *p* < 0.01; *** *p* < 0.001).

In the present study, severe weight loss in STZ treated animals was observed compared to the control group (Table 4). The reduction in body weight may be attributed to insulin depletion provoking a loss of adipose tissues [46]. Intravenous injections of normal rats with STZ induced a statistically significant (*p* < 0.001) increase of the serum levels of glucose (over 350% of basal glycaemia) 72 h post-injection, compared with the control group (group 1) without treatment (Figure 4). Also, significant decrease in plasma insulin (6.97 U/L vs. 15.90 U/L at control group; *p* < 0.01) and total hemoglobin (7.11 mg/dL vs. 14.02 mg/dL at control group; *p* < 0.01) levels was observed in diabetic group as well as significant elevation of glycosylated hemoglobin (HbA_1C_; 12.93% Hb vs. 4.82% Hb at control group; *p* < 0.01). All mentioned changes indicated poor glycemic control and these results were consistent with type 2 diabetes [47].

Oral administration of extract A was significantly increased body weight in diabetic rats compared to STZ-group with maximal level at doze 400 mg/kg. The body weight increase was significantly lower in extract B treated rats than in extract A group. Agrimoniin resulted to the most significant increase of body weight, with more distinct values at a dose of 100 mg/kg, similar to the insulin group data.

The decrease in blood glucose level was dose-dependent and was observed from the seventh day of processing in diabetic rats treated with *C. palustre* herb extracts A and B, agrimoniin and insulin, reaching maximum values at 21 days. In STZ diabetic rats *C. palustre* herb extract A (400 mg/kg, group 5) presented greater hypoglycaemic activity, with a gradual decrease of glycaemia until a maximum reached at 21 days (113 mg/dL). The application of *C. palustre* herb extract B (400 mg/kg, group 8) exhibited low activity in reducing the fasting blood glucose level response, reaching a maximum value of 198 mg/dL at 21 days. The treatment of diabetic rats with agrimoniin solution (100 mg/kg, group 11) showed a considerable reduction of the serum level of glucose at day 14 of the experiment (111 mg/dL).

After 21 days of treatment of diabetic rats with agrimoniin solution at the same concentration, the fasting blood glucose level reduced to 87 mg/dL, which was only 10% higher than in diabetic rats treated with insulin (group 12) and 14% higher than the non-diabetic control rats (group 1). The processing of non-diabetic rats with maximal concentrations of *C. palustre* herb extract A (400 mg/kg, group 13), *C. palustre* herb extract B (400 mg/kg, group 14) and agrimoniin (100 mg/kg, group 15) had no effect on the level of fasting blood glucose within 21 days of the experiment.

The administration of extracts A and B and agrimoniin at increasing concentrations resulted in an increase in the level of plasma insulin in STZ-treated rats. The maximum enhancement in the plasma insulin level was observed in the agrimoniin group at a dose of 100 mg/kg and extract A treated rats received a dose of 400 mg/kg. Treatment with extract A and agrimoniin to diabetic rats significantly increased in a dose-dependent manner the level of total hemoglobin (Hb) and significantly decreased the level of glucosylated hemoglobin (HbA_1c_) when compared to diabetic rats. The effectiveness of extract B was insufficient. Parameters of non-diabetic groups treated with highest dozes of extract A and B (400 mg/kg) as well as agrimoniin (100 mg/kg) were close to the control group demonstrating no influence on healthy animals.

In this way, among the studied samples *C. palustre* extract A and agrimoniin significantly reduced (*p* < 0.001) the serum glucose and normalized the weight and blood parameters (levels of insulin, total and glycosylated hemoglobin) in the STZ diabetic rat model. The antidiabetic effect of *C. palustre* extract B after ellagitannin removal revealed low activity in reducing the fasting blood glucose level response in the same model.

A plausible mechanism for the antidiabetic potential of *C. palustre* extract may be its insulinogenic activity. The observed increase of the plasma level of insulin indicates that *C. palustre* extract stimulates insulin secretion from existing or regenerated β-cells [48]. It should be mentioned that in the process of increased glycation of proteins, including hemoglobin, diabetic complications emerge [49]. The non-enzymatic, irreversible binding of glucose with haemoglobin results in the formation of glycosylated form (HbA_1c_) which was elevated in rats of groups treated with *C. palustre* extract, so it can be concluded that this decrease in the level of HbA_1c_ may be due to the decreased glycosylation of haemoglobin. 

The data obtained confirmed the leading role of agrimoniin in the antidiabetic action of the herb *C. palustre*. Previously it was shown that aqueous extract of *Agrimonia eupatoria* lowered hyperglycaemia in STZ-induced diabetic mice. The mechanism of the hypoglycaemic action of agrimony extract has been investigated by in vitro studies on isolated muscle pieces and consisted of enhanced glucose transport, glucose oxidation, glycogenesis and lactate release comparable with that evoked by 0.01 μM insulin [50]. Granica et al. established that agrimoniin was the dominant compound in aqueous extract of *Agrimonia eupatoria* [51]. These data confirmed that the hypoglycaemic action of agrimoniin dominated in marsh cinquefoil herb as well. In this way, *C. palustre* herb represents a possible dietary adjunct for the treatment of DM, and might be a source of a new oral hypoglycaemic agent. 

## 3. Materials and Methods

### 3.1. Plant Materials and Chemicals

The samples of aerial and subterranean plant organs of *C. palustre* were collected during the flowering and fruiting periods at the Monakhovo Scientific Station (Barguzin District, Republic of Buryatia; 25.VII.2016; 26.VIII.2016; 53°59′26′′ N, 108°89′17′′ E, voucher specimens No. CRo/an-03/23-25/0716 and CRo/an-03/23-26/0816). The species were authenticated by Prof. T.A. Aseeva (IGEB SB RAS, Ulan-Ude). Plant material (root, herb, flowers, seeds) was dried and powdered before analysis. The following chemicals were purchased from Extrasynthese (Lyon, France): quercetin-3-*O*-β-d-glucoside (isoquercitrin; Cat. No. 1327, ≥99%), quercetin-3-*O*-β-d-glucuronide (miquelianin; Cat. No. 1315, ≥95%), quercetin-3-*O*-rutinoside (rutin; Cat. No. 9012, ≥99%); Sigma-Aldrich (St. Louis, MO, USA)—acarbose (Cat. No. A8980, ≥95%), bovine serum albumin (BSA) (Cat. No. 05470, ≥98%), (+)-catechin (Cat. No. 43412, ≥99%), ellagic acid (Cat. No. E2250, ≥95%), gallic acid (Cat. No. G7384, ≥97%), glucose (Cat. No. G8270, ≥99.5%), α-glucosidase from *Saccharomyces cerevisae* (Cat. No. G5003, type 1, 10 U/mg), insulin solution from bovine pancreas (Cat. No. I0516, 10 mg/mL in 25 mM HEPES), kaempferol-3-*O*-α-l-rhamnoside (afzelin; Cat. No. SMB00515, ≥90%), kaempferol-3-*O*-β-rutinoside (nicotiflorin; Cat. No. 90242, ≥98%), kaempferol-3-*O*-β-d-glucopyranoside (astragalin; Cat. No 79851, ≥97%), lithium perchlorate (Cat. No. 431567, ≥99.99%), 4-nitrophenyl-α-d-glucopyranoside (Cat. No. N1377, ≥99%), perchloric acid (Cat. No. 311421, ≥70%, 99.999% trace metals basis), polyamide for CC (Cat. No. 02395), procyanidin B_3_ (Cat. No. P1066, ≥80%), sodium carbonate (Cat. No. 791768, ≥99.5%), streptozotocin (Cat. No. S0130, ≥98%); Chemwill Asia Co., Ltd. (Beijing, China)—agrimoniin (Cat. No. 82203). 2-Pyrone-4,6-dicarboxylic acid, α-pedunculagin, β-pedunculagin, and potentillin were isolated previously from *C. palustre* [21]. Equipment used for UV-Vis spectrophotometry was a SF-2000 UV-Vis-spectrophotometer (OKB Specter, St. Peterburg, Russia); analytical MC-HPLC—was performed on a MiLiChrom A-02 microcolumn chromatograph (Econova, Novosibirsk, Russia).

### 3.2. Sample Preparation

For preparation of decoctions, accurately weighed *C. palustre* plant samples (1 g) were placed in conical flasks. Then 100 mL of distilled water was added and the samples were heated on a hotplate and boiled for 10 min. The mixture was left to stand at room temperature for 15 min, and then filtered under reduced pressure. For preparation of infusions, accurately weighed *C. palustre* plant samples (1 g) were placed in conical flasks. Then 100 mL of boiled distilled water was added. The sample was then stirred for 40 min. Then the mixture was filtered under reduced pressure. For preparation of tinctures, accurately weighed *C. palustre* plant samples (10 g) were placed in conical flasks. Then 100 mL of 40% ethanol solution was added. The mixture was left to stand at room temperature for 7 days, and then filtered under reduced pressure. For preparation of ethanol extracts (30%, 60%, 96%), accurately weighed *C. palustre* plant samples (100 g) were placed in conical flasks. Then 1500 mL of the appropriate ethanol solution (30%, 60%, 96%) was added and mixture was extracted twice in an ultrasonic bath for 90 min at 45 °C. The extracted solutions were filtered through a cellulose filter and evaporated *in vacuo* until dryness using a rotary evaporator.

### 3.3. MC-RP-HPLC-UV-MS and MC-HPLC-UV Conditions 

MC-RP-HPLC-UV-MS experiments were performed on an Econova MiLiChrom A-02 microcolumn chromatograph (Novosibirsk, Novosibirsk Oblast, Russia) coupled with UV- and ESI-MS-detectors, using a ProntoSIL-120-5-C18 AQ column (1 × 50 mm, Ø 1 μm; Metrohm AG, Herisau, Switzerland); the column temperature was 35 °C. Eluent A was 0.2 M LiClO_4_ in 0.006 M HClO_4_ and eluent B was acetonitrile. The injection volume was 1 μL, and elution was at 300 μL/min. Gradient program: 0–2.5 min 11%–18% B, 2.5–4.5 min 18% B, 4.5–5.5 min 18%–20% B, 5.5–6.5 min 20%–25% B, 6.5–8.0 min 25% B, 8.0–10.0 min 25%–100% B, 10.0–15.0 min 100% B. The column was equilibrated 1 min between injections. UV spectra were recorded in the range of 200–400 nm. Chromatograms were acquired at 270 nm. A 6200 TOF LC/MS TOF mass spectrometer (Agilent, Santa Clara, CA, USA) with an ESI interface was used. The parameters of the ESI source were: nebulizer pressure 40 psi; nebulizing gas and drying gas were nitrogen at a flow 10 L∙min^−1^; dry temperature 325 °C; capillary voltage −3.5 kV. Analysis was carried out using scans from *m*/*z* 150–2000. For preparation of dry extract solution, an accurately weighed dry extract of *C. palustre* (10 mg) was placed in an Eppendorf tube, 1 mL of 60% ethanol was added, and the mixture was weighed again. Then the sample was extracted in an ultrasonic bath for 10 min at 40 °C. After cooling, the tube weight was reduced to initial sign, and the resultant extract was filtered through a 0.22-μm PTFE syringe filter before injection into the HPLC system for analysis. 

MC-HPLC-UV quantification experiments were carried out at the same chromatographic conditions with UV-detection at 270 nm. Stock solutions of standards were made by accurately weighing 1 mg samples of 2-pyrone-4,6-dicarboxylic acid, gallic acid, α-pedunculagin, β-pedunculagin, procyanidin B3, (+)-catechin, potentillin, agrimoniin, ellagic acid, quercetin-3-*O*-rutinoside (rutin), quercetin-3-*O*-β-d-glucuronide (miquelianin), quercetin-3-*O*-β-d-glucoside (isoquercitrin), kaempferol-3-*O*-β-rutinoside (nicotiflorin), kaempferol-3-*O*-β-d-glucopyranoside (astragalin) and kaempferol-3-*O*-α-l-rhamnoside (afzelin), and dissolving them in 20 mL of methanol/DMSO in a volumetric flask. The appropriate amounts of stock solutions were diluted with methanol in order to obtain standard solutions containing 0.25–1.00 mg/mL. As all the compounds used for quantification were well-separated under the experimental conditions, mixtures of standards were analyzed. Prepared solutions were stored at 4 °C for no more than 72 h. The results are presented as mean values ± SD (standard deviations) of three replicates.

### 3.4. HPLC Activity-Based Profiling

HPLC activity-based profiling was realized using post-chromatographic reaction of HPLC-eluates with α-glucosidase. Aliquots (100 μL) of extract soln. (10 mg/mL) was separated under analytical HPLC conditions (Section 3.4). The eluates (50 μL) were collected every 30 sec using an automated fraction collector (Econova) in 96-well plates, then dried under a N_2_-steam, redissolved in 10 μL of phosphate buffer (pH 6.8) and analyzed according to a published method [52]. α-Glucosidase from *Saccharomyces cerevisiae* was dissolved in phosphate buffer (pH 6.8) containing BSA (0.2%) up to 0.5 U/mL concentration. Aliquots (125 μL) of phosphate buffer (pH 6.8) and 60 μL 5 mM *p*-nitrophenyl-α-d-glucopyranoside were added into all wells. After preincubating at 37 °C for 5 min, 60 μL α-glucosidase (0.4 U/mL) was added and incubated at 37 °C for 15 min. The reaction was terminated by the addition of 50 μL Na_2_CO_3_ (200 mM). Absorbance was measured at 400 nm. (A_400_). The results were performed as (1-A_400_) to create HPLC-based bioactivity profiles of the extract. The (1-A_400_) values were plotted under the corresponding chromatogram section and the higher value of (1-A_400_) showed the better anti-α-glucosidase activity of the fraction.

### 3.5. Ellagitannin Remove SPE-Procedure

A sample of 60% ethanol extract of *C. palustre* herb (1 g; extract A, untreated) was dissolved in 100 mL of 40% ethanol and 100 mL of water was additionally added. A polyamide column (20 g) was prepared and primed with 150 mL methanol followed by 200 mL tridistilled water (td-water). An aliquot (200 mL) of 60% ethanol extract of *C. palustre* herb was loaded on the polyamide column. Sequential elution was done with 300 mL of td-water and 400 mL of 70% ethanol. The latter fraction after SPE extraction was evaporated *in vacuo* until dryness using a rotary evaporator. The yield of de-tannified extract (extract B) was 37% of the untreated extract weight.

### 3.6. α-Glucosidase Inhibiting Assay

The α-glucosidase inhibition assay was performed using a spectrophotometric method [19]. α-Glucosidase from *Saccharomyces cerevisiae* was dissolved in phosphate buffer (pH 6.8) containing BSA (0.2%) up to 0.5 U/mL concentration. Solutions (10 μL) of sample in phosphate buffer (pH 6.8) of varying concentrations (10–1000 μg/mL) were premixed with 490 μL of phosphate buffer (pH 6.8) and 250 μL 5 mM *p*-nitrophenyl-α-d-glucopyranoside. After preincubating at 37 °C for 5 min, 250 μL of α-glucosidase (0.4 U/mL) was added and incubated at 37 °C for 15 min. The reaction was terminated by the addition of 200 μL Na_2_CO_3_ (200 mM). Absorbance was measured at 400 nm. A 2% solution of acarbose was used as a positive control (PC), and water was used as a negative control (NC). The ability to inhibit glucosidase was calculated using the following equation:

Inhibitory ability (%) = [(A_400_^NC^ − A_400_^PC^) − (A_400_^Sample^ − A_400_^PC^)/(A_400_^NC^ − A_400_^PC^)] × 100,

where A_400_^NC^ is the absorbance of the negative control, A_400_^PC^ is the absorbance of the positive control and A_400_^Sample^ is the absorbance of the sample solution. The IC_50_ value is the effective concentration at which α-glucosidase activity was inhibited by 50%. Values are expressed as mean from five independent experiments.

### 3.7. Hypoglycemic Activity

#### 3.7.1. Experimental Animals

Experiments were performed on adult male Wistar rats (body weight range: 180–210 g), 8 to 10 weeks of age, obtained from the ‘Pushchino’ Laboratory Animal Breeding House, Moscow (Russia), 2–3 days before the start of experiment. Animals were housed and maintained at 22 °C under a 12/12 light/dark cycle, with free access to food and water. Experiments were carried out during the normal light/dark cycle and always started at the same hour (10 a.m.). The animals had access to food and water ad libitum. Efforts were made to minimize animal suffering and to reduce the number of animals used. The experimental procedures relating to the animals were authorised by the Institute of General and Experimental Biology’s Ethical Committee (protocol No. LM-0324, 27 January 2012) before starting the study and were conducted under the internationally accepted principles for laboratory animal use and care.

#### 3.7.2. Hyperglycemia Induction and Experimental Design

Experimental diabetes [53] was induced following an overnight fast by a single intraperitoneal injection of 60 mg/kg streptozotocin (Sigma, St. Louis, MO, USA) freshly dissolved in distilled water. Since streptozotocin is capable of producing fatal hypoglycemia as a result of massive pancreatic release of insulin, the rats were treated with 20% glucose solution intraperitoneally after 6 h [54]. They were kept for the next 24 h on 5% glucose soln. bottles in their cages to prevent hypoglycemia. Hyperglycemia was confirmed four days after injection by measuring the tail vein blood glucose level with an Accu-Check Sensor Comfort Glucometer (Roche, Mexico City, Mexico). The rats with a blood glucose levels greater than ≥250 mg/dL were considered diabetic and used for this research work. Control animals received 0.9% sterile saline. Insulin-treated diabetic rats served as positive control [55]. Fasting blood glucose levels of all the rats were determined before the start of the experiment. Rats were divided into the following groups: (1) control group given only saline (300 μL/kg/once a day, daily); streptozotocin induced diabetic rats given in (2) saline (10 mg/kg/day); (3) *C. palustre* herb extract A (100 mg/kg/day); (4) *C. palustre* herb extract A (200 mg/kg/day); (5) *C. palustre* herb extract A (400 mg/kg/day); (6) *C. palustre* herb extract B (100 mg/kg/day); (7) *C. palustre* herb extract B (200 mg/kg/day); (8) *C. palustre* herb extract B (400 mg/kg/day); (9) agrimoniin (25 mg/kg/day); (10) agrimoniin (50 mg/kg/day); (11) agrimoniin (100 mg/kg/day); (12) insulin (5 U/kg/day); non-diabetic rats treated with (13) *C. palustre* herb extract A (400 mg/kg/day); (14) *C. palustre* herb extract B (400 mg/kg/day); (15) agrimoniin (100 mg/kg/day). The study extracts and agrimoniin were dissolved in saline and administered daily to rats by gastric oral tube and insulin was given subcutaneous. The study period was carried out for 21 days, fasting blood glucose of the animals was determined on 1, 7, 14 and 21 days. Plasma insulin was determined by an Enzyme-linked immunosorbent assay (rat/mouse Insulin ELISA kit, Linco Research, Inc.; St. Charles, MO, USA). Total hemoglobin and glycosylated hemoglobin A_1c_ (HbA_1c_) was separate by a chromatographic method using a cationic exchange resin (Hemoglobin A_1c_ Kit, BioSystem S.A., Barcelona, Spain) and quantified by spectrophotometric reading at 415 nm [56].

### 3.8. Statistical Analysis

Statistical analyses were performed using a one-way analysis of variance (ANOVA), and the significance of the mean difference was determined by Duncan’s multiple range test. Differences at *p* < 0.05 were considered statistically significant. The results are presented as mean values ± SD (standard deviations) of the three replicates.

## 4. Conclusions

In this study, we investigated the anti-α-glucosidase and antidiabetic potential of two 60% ethanol extracts from *C. palustre* herb (before and after ellagitannin removal) and agrimoniin as the most active compound of the extracts, in STZ-induced diabetic rats. Due to the high inhibitory activity against α-glucosidase of the extract, its component profile was studied and it was screened for potential inhibitors using HPLC activity-based profiling. Since agrimoniin revealed the highest activity in relation to α-glucosidase inhibition, the removal of ellagitannins from the extract was realized to confirm its activity. Using in vivo experiments the antidiabetic effect of extracts before ellagitannin removal was demonstrated and proved that agrimoniin was responsible for the observed hypoglycaemic activity of the *C. palustre* herb extract. The application of agrimoniin significantly reduced the level of plasma glucose and glycosylated hemoglobin and significantly increased the value of plasma insulin and total hemoglobin in the treated diabetic groups compared to the diabetic control group. The results demonstrate the benefits of application of the *C. palustre* herb extract and agrimoniin as prospective substances for the treatment of DM.

## Figures and Tables

**Table 1:** Inhibitory effect on α-glucosidase of *Comarum palustre* extracts (IC_50_, μg/mL) ^a,b^.

Plant Part	Decoction	Infusion	Tincture	30% Ethanol Extract	60% Ethanol Extract	96% Ethanol Extract
Herb	127.4 ± 5.3 ^iii^	142.9 ± 5.8 ^iii^	98.4 ± 3.7 ^ii^	89.4 ± 3.3 ^i,ii^	52.0 ± 1.7 ^i^	154.7 ± 6.0 ^iii,iv^
Roots	>300	>300	>300	>300	272.7 ± 10.6 ^v^	>300
Flowers	>300	>300	254.9 ± 9.9 ^iv,v^	183.3 ± 7.1 ^iv^	104.2 ± 4.3 ^iii^	>300
Seeds	>300	>300	>300	>300	>300	>300

^a^ Average of three analyses (± standard deviation (SD)); ^b^ acarbose was used as a reference compound with IC_50_ value 294.4 ± 11.4 μg/mL. All values correspond to mean values ± SD of three replicates. Values with different letters (i–v) indicate statistically significant differences among groups at *p* < 0.05 by one-way ANOVA.

**Table 2:** MC-RP-HPLC-UV-MS characterization of the components of *C. palustre* extract.

Peak No.	Compound	*t*_R,min_	λ_max,nm_	ESI-MS, *m*/*z*	Method ^a^
1	2-Pyrone-4,6-dicarboxylic acid	0.75	212, 316	138 [M − H]^−^	i, ii, iii
2	Gallic acid	0.92	220, 270	169 [M − H]^−^	i*, ii*, iii*
3	α-Pedunculagin	2.53	235	783 [M − H]^−^	i, ii, iii
4	β-Pedunculagin	3.31	235	783 [M − H]^−^	i, ii, iii
5	Procyanidin B_3_	3.80	240, 278	577 [M − H]^−^	i*, ii*, iii*
6	(+)-Catechin	4.15	240, 278	289 [M − H]^−^	i*, ii*, iii*
7	Potentillin	6.21	220, 256	935 [M − H]^−^	i, ii, iii
8	Agrimoniin	6.98	228, 270	1869 [M − H]^−^, 934 [M − 2H]^2−^	i*, ii*, iii*
9	Ellagic acid	7.20	250, 367	301 [M − H]^−^, 603 [2M − H]−	i*, ii*, iii*
10	Rutin	7.33	258, 356	609 [M − H]^−^, 301 [M − H − Rut]^−^	i*, ii*, iii*
11	Miquelianin	7.50	254, 355	477 [M − H]^−^, 301 [M − H − GlcA]^−^	i*, ii*, iii*
12	Isoquercitrin	7.72	254, 356	463 [M − H]^−^, 301 [M − H − Glc]^−^	i*, ii*, iii*
13	Nicotiflorin	8.12	364, 350	593 [M − H]^−^, 285 [M − H − Rut]^−^	i*, ii*, iii*
14	Astragalin	8.55	265, 350	447 [M − H]^−^, 285 [M − H − Glc]^−^	i*, ii*, iii*
15	Afzelin	9.24	265, 351	431 [M − H]^−^, 285 [M − H − Rha]^−^	i*, ii*, iii*

^a^ Identification method: comparing of retention time (i); UV- (ii); and MS-spectra (iii) with those of isolated compounds (no asterisk) or commercial standards (*). Glc: glucose; GlcA: glucuronic acid; Rha: rhamnose; Rut: rutinose.

**Table 3:** Quantification of the components of *C. palustre* herb extracts before (extract A) and after ellagitannin removal (extract B).

HPLC Peak No.	Compound	Extract A, mg·g^−1 a^	Extract B, mg·g^−1 a^
1	2-Pyrone-4,6-dicarboxylic acid	3.25 ± 0.06	4.52 ± 0.11
2	Gallic acid	0.50 ± 0.01	n.d.
3	α-Pedunculagin	9.80 ± 0.25	n.d.
4	β-Pedunculagin	10.79 ± 0.33	n.d.
5	Procyanidin B_3_	30.02 ± 0.78	0.52 ± 0.01
6	(+)-Catechin	28.02 ± 0.67	n.d.
7	Potentillin	22.82 ± 0.66	n.d.
8	Agrimoniin	240.94 ± 6.74	1.03 ± 0.02
9	Ellagic acid	6.67 ± 0.18	n.d.
10	Rutin	8.07 ± 0.21	12.20 ± 0.32
11	Miquelianin	80.81 ± 2.10	120.27 ± 3.72
12	Isoquercitrin	8.67 ± 0.25	12.95 ± 0.33
13	Nicotiflorin	6.45 ± 0.18	9.82 ± 0.26
14	Astragalin	25.40 ± 0.71	32.11 ± 0.99
15	Afzelin	6.35 ± 0.17	9.64 ± 0.26
Total ellagitannins (Σ_3,4,7,8_)		284.35	1.03
Total flavonoids (Σ_10–15_)		135.75	196.99
Total catechines (Σ_5,6_)		58.04	0.52
Other classes (Σ_1,2,9_)		10.42	4.52
Total phenolics (Σ_1–15_)		488.56	203.06

^a^ Average of three analyses (±SD).

**Table 4:** Parameters of body weight, insulin, total hemoglobin (Hb) and glycosylated hemoglobin (HbA_1c_) of control and experimental groups, mean ± SD.

Experimental group	Body Weight, g		Insulin, U/L	Hb, mg/dL	HbA_1c_, %Hb
	0 Day	21 Day			
Control	195 ± 6	232 ± 7	15.90 ± 1.23	14.02 ± 0.84	4.82 ± 0.28
STZ + saline	198 ± 5	121 ± 6 ^a^	6.97 ± 0.41 ^a^	7.11 ± 0.43 ^a^	12.93 ± 0.77 ^a^
STZ + Extract A (100 mg/kg)	192 ± 4	149 ± 5 ^a,c^	7.35 ± 0.44 ^a^	8.39 ± 0.41 ^a,c^	12.52 ± 0.62 ^a^
STZ + Extract A (200 mg/kg)	192 ± 4	151 ± 5 ^a,c^	9.14 ± 0.59 ^a,d^	9.63 ± 0.48 ^a,c^	10.83 ± 0.54 ^a,c^
STZ + Extract A (400 mg/kg)	199 ± 6	197 ± 7 ^b,d^	14.05 ± 0.91 ^d^	12.92 ± 0.64 ^d^	7.22 ± 0.36 ^b,d^
STZ + Extract B (100 mg/kg)	190 ± 3	120 ± 3 ^a^	6.90 ± 0.40 ^a^	7.32 ± 0.36 ^a^	12.70 ± 0.60 ^a^
STZ + Extract B (200 mg/kg)	192 ± 4	127 ± 5 ^a^	7.04 ± 0.47 ^a^	7.55 ± 0.39 ^a^	12.34 ± 0.58 ^a^
STZ + Extract B (400 mg/kg)	195 ± 4	138 ± 4 ^a,c^	7.37 ± 0.42 ^a^	8.39 ± 0.41 ^a^	11.25 ± 0.56 ^a^
STZ + Agrimoniin (25 mg/kg)	193 ± 5	183 ± 3 ^b,d^	10.35 ± 0.70 ^ad^	8.56 ± 0.40 ^a,d^	11.38 ± 0.56 ^a^
STZ + Agrimoniin (50 mg/kg)	198 ± 7	202 ± 5 ^b,d^	14.10 ± 0.91 ^d^	10.81 ± 0.52 ^b,d^	9.06 ± 0.42 ^b,c^
STZ + Agrimoniin (100 mg/kg)	195 ± 3	219 ± 7 ^d^	15.22 ± 1.06 ^d^	13.26 ± 0.66 ^d^	6.14 ± 0.30 ^b^
STZ + Insulin	192 ± 4	228 ± 8 ^d^	15.63 ± 0.92 ^d^	13.73 ± 0.68 ^d^	5.11 ± 0.25 ^d^
Extract A (400 mg/kg)	195 ± 5	236 ± 7 ^d^	15.97 ± 1.11 ^d^	14.19 ± 0.73 ^d^	4.93 ± 0.23 ^d^
Extract B (400 mg/kg)	192 ± 5	243 ± 9 ^d^	15.86 ± 1.02 ^d^	14.11 ± 0.70 ^d^	4.87 ± 0.24 ^d^
Agrimoniin (100 mg/kg)	194 ± 4	230 ± 8 ^d^	15.90 ± 1.09 ^d^	14.06 ± 0.71 ^d^	4.80 ± 0.20 ^d^

Letters a, b indicate statistically significant values from control group (^a^ *p* < 0.01; ^b^ *p* < 0.05). Letters c, d indicate statistically significant values from STZ-group (^c^ *p* < 0.01; ^d^ *p* < 0.05). STZ: streptozotocin.

## References

[1] World Health Organization (2016). Global Report on Diabetes.

[2] Ríos J.L., Francini F., Schinella G.R. (2015). Natural products for the treatment of type 2 diabetes mellitus. Planta Med..

[3] Scheen A.J. (2003). Is there a role for α-glucosidase inhibitors in the prevention of type 2 diabetes mellitus?. Drugs.

[4] Kihara Y., Ogami Y., Tabaru A., Unoki H., Otsuki M. (1997). Safe and effective treatment of diabetes mellitus associated with chronic liver diseases with an alpha-glucosidase inhibitor, acarbose. J. Gastroenterol..

[5] Güemes M., Melikyan M., Senniappan S., Hussain K. (2016). Idiopathic postprandial hyperinsulinic hypoglycaemia. J. Pediatr. Endocrinol. Metab..

[6] Gray A.M., Flatt P.R. (1997). Nature’s own pharmacy: The diabetes perspective. Proc. Nutr. Soc..

[7] Xiao J., Kai G., Yamamoto K., Chen X. (2013). Advance in dietary polyphenols as α-glucosidases inhibitors: A review on structure-activity relationship aspect. Crit. Rev. Food Sci. Nutr..

[8] Zhang J., Zhao S., Yin P., Yan L., Han J., Shi L., Zhou X., Liu Y., Ma C. (2014). α-Glucosidase inhibitory activity of polyphenols from the burs of *Castanea mollissima* Blume. Molecules.

[9] Yuan T., Wan C., Ma H., Seeram N. (2013). New phenolics from the flowers of *Punica granatum* and their in vitro α-glucosidase inhibitory activities. Planta Med..

[10] Flores-Bocanegra L., Pérez-Vásquez A., Torres-Piedra M., Bye R., Linares E., Mata R. (2015). α-Glucosidase inhibitors from *Vauquelinia corymbosa*. Molecules.

[11] Shaheen K., Itrat F., Azhar M., Rehana A., Abdul M., Sumaira T., Muhammad I.C. (2009). Purunusides A-C, α-glucosidase inhibitory homoisoflavone glucosides from *Prunus domestica*. Arch. Pharm. Res..

[12] Šaponjak V.T., Gironés-Vilaplana A., Djilas S., Mena P., Ćetković G., Moreno D.A., Ćanadanović-Brunet J., Vulić J., Stajćić S., Krunić M. (2014). Anthocyanin profiles and biological properties of caneberry (*Rubus* spp.) press residues. J. Sci. Food Agric..

[13] Yin Z.H., Wang J.J., Gu X.Z., Gul H.P., Kang W.Y. (2012). Antioxidant and a-glucosidase inhibitory activity of red raspberry (Harrywaters) fruits in vitro. Afr. J. Pharm. Pharmacol..

[14] Rusakova L.M. (1983). Traditions and Innovations in Life and Culture of the Siberian Peoples.

[15] Makarov A.A. (1974). Plant Remedies of the Traditional Yakutian Medicine.

[16] Popov S.V., Popova G.Y., Ovodova R.G., Ovodov Y.S. (2005). Antiinflammatory activity of the pectic polysaccharide from *Comarum palustre*. Fitoterapia.

[17] Popov S.V., Ovodova R.G., Markov P.A., Nikitina I.R., Ovodov Y.S. (2006). Protective effects of comaruman, a pectin of cinquefoil *Comarum palustre* L. on acetic acid-induced colitis in mice. Dig. Dis. Sci..

[18] Lemus I., García R., Delvillar E., Knop G. (1999). Hypoglycaemic activity of four plants used in Chilean popular medicine. Phytother. Res..

[19] Elya B., Basah K., Mun’im A., Yuliastuti W., Bangun A., Septiana E.K. (2012). Screening of α-Glucosidase inhibitory activity from some plants of Apocynaceae, Clusiaceae, Euphorbiaceae, and Rubiaceae. J. Biomed. Biotechnol..

[20] Pinto M.D., Ranilla L.G., Apostolidis E., Lajolo F.M., Genovese M.I., Shetty K. (2009). Evaluation of antihyperglycemia and antihypertension potential of native Peruvian fruits using in vitro models. J. Med. Food.

[21] Olennikov D.N. (2016). Ellagitannins and other phenolic compounds from *Comarum palustre*. Chem. Nat. Comp..

[22] Julianti T., Mieri M.D., Zimmermann S., Ebrahimi S.N., Kaiser M., Neuburger M., Raith M., Brun R., Hamburger M. (2014). HPLC-based activity profiling for antiplasmodial compounds in the traditional Indonesian medicinal plant *Carica papaya* L. J. Ethnopharmacol..

[23] Yang X., Baburin I., Plitzko I., Hering S., Hamburger M. (2011). HPLC-based activity profiling for GABA_a_ receptor modulators from the traditional Chinese herbal drug Kushen (*Sophora flavescens* root). Mol. Divers..

[24] Dittmann K., Riese U., Hamburger M. (2004). HPLC-based bioactivity profiling of plant extracts: A kinetic assay for the identification of monoamine oxidase-A inhibitors using human recombinant monoamine oxidase-A. Phytochemistry.

[25] Potterat O., Hamburger M. (2006). Natural products in drug discovery—Concepts and approaches for tracking bioactivity. Curr. Org. Chem..

[26] Yuan T., Ding Y., Wan C., Li L., Xu J., Liu K., Slitt A., Ferreira D., Khan I.A., Seeram N.P. (2012). Antidiabetic ellagitannins from pomegranate flowers: Inhibition of α-glucosidase and lipogenic gene expression. Org. Lett..

[27] Santos-Buelga C., Scalbert A. (2000). Proanthocyanidins and tannin-like compounds—Nature, occurrence, dietary intake and effects on nutrition and health. J. Sci. Food Agric..

[28] Ochir S., Nishizawa M., Park J.B., Yamagashi T. (2010). Inhibitory effects of *Rosa gallica* on the digestive enzymes. J. Nat. Med..

[29] Bellesia A., Verzelloni E., Taqliazucchi D. (2015). Pomegranate ellagitannins inhibit α-glucosidase activity in vitro and reduce starch digestibility under simulated gastro-intestinal conditions. Int. J. Food Sci. Nutr..

[30] Yin Z., Zhang W., Feng F., Zhang Y., Kang W. (2014). α-Glucosidase inhibitors isolated from medicinal plants. Food Sci. Hum. Wellness.

[31] McDougall G.J., Shpiro F., Dobson P., Smith P., Blake A., Stewart D. (2005). Different polyphenolic components of soft fruits inhibit alpha-amylase and alpha-glucosidase. J. Agric. Food Chem..

[32] Xiao J., Ni X., Kai G., Chen X. (2013). A review on structure-activity relationship of dietary polyphenols inhibiting α-amylase. Crit. Rev. Food Sci. Nutr..

[33] Wang H., Du Y.-J., Song H.-C. (2010). α-Glucosidase and α-amylase inhibitory activities of guava leaves. Food Chem..

[34] Li Y.Q., Zhou F.C., Gao F., Bian J.S., Shan F. (2009). Comparative evaluation of quercetin, isoquercetin and rutin as inhibitors of α-glucosidase. J. Agric. Food Chem..

[35] Kim J., Kwon C., Son K. (2000). Inhibition of alpha-glucosidase and amylase by luteolin, a flavonoid. Biosci. Biotehnol. Biochem..

[36] Zhou H., Xing J., Liu S., Song F., Cai Z., Pi Z., Liua Z., Liua S. (2012). Screening and determination for potential α-glucosidase inhibitors from leaves of *Acanthopanax senticosus* Harms by using UF-LC/MS and ESI-MS^n^. Phytochem. Anal..

[37] Toda M., Kawabata J., Kasai T. (2001). Inhibitory effects of ellagi- and gallotannins on rat intestinal α-Glucosidase complexes. Biosci. Biotech. Biochem..

[38] Spencer C.M., Cai Y., Martin R., Gaffney S.H., Goulding P.N., Mangnolato D., Lilley Y., Haslam E. (1988). Polyphenol complexation—Some thoughts and observatios. Phytochemistry.

[39] Bai N., He K., Roller M., Zheng B., Chen X., Shao Z., Peng T., Zheng Q. (2008). Active compounds from *Lagerstroemia speciosa*, insulin-like glucose uptake-stimulatory/inhibitory and adipocyte differentiation-inhibitory activities in 2T3-L1 cells. J. Agric. Food Chem..

[40] Liu X., Kim J.K., Li Y., Li J., Liu F., Chen X. (2005). Tannic acid stimulates glucose transport and inhibits adipocyte differentiation in 3T3-L1 cells. J. Nutr..

[41] Broadhurst C.L., Polansky M.M., Anderson R.A. (2000). Insulin-like biological activity of culinary and medicinal plant aqueous extracts in vitro. J. Agric. Food Chem..

[42] Juśkiewicz J., Jurgonski A., Ko󠅲łodziejczyk K., Kosmala M., Milana J., Zduńczyk Z., Fotschki B., Zary-Sikorska E. (2016). Blood glucose lowering efficacy of strawberry extracts rich in ellagitannins with different degree of polymerization in rats. Pol. J. Food Nutr. Sci..

[43] Gomes J.R., Vedasiromoni M.D., Sharma R.M., Ganguly D.K. (2001). Antihyperglycemic effect of black tea (*Camellia sinensis*) in rat. J. Ethnopharmacol..

[44] Mohamed A.K., Bierhaus A., Schiekofer S., Tritschler H., Ziegler R., Nawroth P.P. (1999). The role of oxidative stress and NF-κB activation in late diabetic complications. BioFactors.

[45] Weir G.C., Clore E.T., Zmachiroski C.J., Bonner-Weir S. (1981). Islet secretion in a new experiment model for non-insulin dependent diabetes. Diabetes.

[46] Saravanan R., Ramachandran V. (2012). Effect of rebaudioside A, a diterpenoid on glucose homeostasis in STZ-induced diabetic rats. J. Physiol. Biochem..

[47] Lenzen S. (2008). The mechanisms of alloxan- and streptozotocin-induced diabetes. Diabetologia.

[48] Pareek H., Sharma S., Khajja B.S., Jain K., Jain G.C. (2009). Evaluation of hypoglycemic and anti-hyperglycemic potential of *Tridax procumbens* (Linn.). BMC Complement. Altern. Med..

[49] Ahmed D., Sharma M., Kumar V., Bajaj H.K., Verma A. (2015). 2β-hydroxybetulinic acid 3β-caprylate: An active principle from *Euryale ferox* Salisb. seeds with antidiabetic, antioxidant, pancreas & hepatoprotective potential in streptozotocin induced diabetic rats. J. Food Sci. Technol..

[50] Gray A.M., Flatt P.R. (1998). Actions of the traditional anti-diabetic plant, *Agrimony eupatoria* (agrimony): Effects on hyperglycaemia, cellular glucose metabolism and insulin secretion. Br. J. Nutr..

[51] Granica S., Krupa K., Kłębowska A., Kiss A.K. (2013). Development and validation of HPLC-DAD-CAD-MS^3^ method for qualitative and quantitative standardization of polyphenols in *Agrimoniae eupatoriae* herba (Ph. Eur). J. Pharm. Biomed. Anal..

[52] Olennikov D.N., Kashchenko N.I. (2014). Compotential profile and amylase inhibiting activity of phenolic compounds from *Calendula officinalis* L. leaves. Sci. World J..

[53] Courteix C., Bardin M., Chantelauze C., Lavarenne J., Eschalier A. (1994). Study of the sensitivity of the diabetes-induced pain model in rats to a range of analgesics. Pain.

[54] Stanley A., Mainzen P., Venugopal M.P. (2001). Anti-oxidant action of *Tinospora cordifolia* root extract in alloxan diabetic rats. Phytother. Res..

[55] Gupta S., Kataria M., Gupta P.K., Murganandan S., Yashroy R.C. (2004). Protective role of extracts of neem seeds in diabetes caused by streptozotocin in rats. J. Ethnopharmacol..

[56] Serra-Barcellona C., Coll Aráoz M.V., Cabrera W.M., Habib N.C., Honoré S.M., Catalán C.A.N., Grau A., Genta S.B., Sánchez S.S. (2014). *Smallanthus macroscyphus*: A new source of antidiabetic compounds. Chem. Biol. Interact..

